# Analysis of the Physiological and Molecular Responses of *Dunaliella salina* to Macronutrient Deprivation

**DOI:** 10.1371/journal.pone.0152226

**Published:** 2016-03-29

**Authors:** Hexin Lv, Xianggan Cui, Fazli Wahid, Feng Xia, Cheng Zhong, Shiru Jia

**Affiliations:** 1 Key Laboratory of Industrial Fermentation Microbiology, Ministry of Education, Tianjin University of Science and Technology, Tianjin, P. R. China; 2 Tianjin Key Lab of Industrial Microbiology, Tianjin University of Science and Technology, Tianjin, P. R. China; Zhejiang University, CHINA

## Abstract

The halotolerant chlorophyte *Dunaliella salina* can accumulate up to 10% of its dry weight as *β*-carotene in chloroplasts when subjected to adverse conditions, including nutrient deprivation. However, the mechanisms of carotenoid biosynthesis are poorly understood. Here, the physiological and molecular responses to the deprivation of nitrogen (-N), sulfur (-S), phosphorus (-P) and different combinations of those nutrients (-N-P, -N-S, -P-S and -N-P-S) were compared to gain insights into the underlying regulatory mechanisms of carotenoid biosynthesis. The results showed that both the growth and photosynthetic rates of cells were decreased during nutrient deprivation, accompanied by lipid globule accumulation and reduced chlorophyll levels. The SOD and CAT activities of the cells were altered during nutrient deprivation, but their responses were different. The total carotenoid contents of cells subjected to multiple nutrient deprivation were higher than those of cells subjected to single nutrient deprivation and non-stressed cells. The *β*-carotene contents of cells subjected to -N-P, -N-S and -N-P-S were higher than those of cells subjected to single nutrient deprivation. Cells subjected to sulfur deprivation accumulated more lutein than cells subjected to nitrogen and phosphorous deprivation. In contrast, no cumulative effects of nutrient deprivation on the transcription of genes in the carotenogenic pathway were observed because MEP and carotenogenic pathway genes were up-regulated during single nutrient deprivation but were downregulated during multiple nutrient deprivation. Therefore, we proposed that the carotenoid biosynthesis pathway of *D*. *salina* is regulated at both the transcriptional and posttranscriptional levels and that a complex crosstalk occurs at the physiological and molecular levels in response to the deprivation of different nutrients.

## Introduction

Carotenoids are derived from two isoprene isomers: dimethylallyl diphosphate (DMAPP) and isopentenyl diphosphate (IPP). The IPP and DMAPP that are used for carotenoid biosynthesis in microalgae are derived from the methylerythritol 4-phosphate (MEP) pathway. MEP is formed via reduction of DXP by DXP reductoisomerase (DXR) [1]. Two rate-determining enzymes in MEP pathway have been identified. 1-deoxy-D-xylulose 5-phosphate synthase (DXS) is involved in the initial step, while 4-hydroxy-3-methylbut-2-enyl diphosphate reductase (HDR) is responsible for converting 4-hydroxy-3-methylbut-2-enyl diphosphate (HMBPP) to DMAPP and IPP [2]. Phytoene synthase (PSY) catalyzes the head to head combination of two molecules of GGPP, resulting in the generation of phytoene. PSY has been regarded as a key enzyme in carotenogenesis, and it may be the regulatory point that determines the flux of carbon towards carotenoids [3]. The next steps in carotenogenesis after phytoene biosynthesis are the stepwise desaturation reactions that result in the conversion of phytoene to lycopene via phytofluene, *ζ*-carotene, and neurosporene as intermediates. In plants, these dehydrogenation reactions are achieved by two desaturases, phytoene desaturase (PDS) and *ζ*-carotene desaturase (ZDS), which catalyze two sequential dehydrogenation reactions and add two symmetrical double bonds, respectively [4]. Following ring formation by cyclases (lycopene *β*-cyclase and / or lycopene *ε*-cyclase) the synthesis of *α*- and *β*-carotene and further hydroxylation reactions by hydrolase result in the formation of lutein or violanxanthin, from which other end-product carotenoids can be formed [5].

The green alga *Dunaliella salina* can accumulate high amount of *β*-carotene which is more than 14% of its dry weight in its cup shaped chloroplast when subjected to abiotic stresses. *β*-carotene and lutein are major carotenoids of *D*. *salina* and account for 90% and 5% of total carotenoids, respectively [6]. *D*. *salina* has been regarded as a valuable model for understanding the regulation of carotenogenesis [7, 8]. Carotenogenesis in *D*. *salina* depends on the supply of MEP-derived precursors [9, 10]. To date, many genes that encode enzymes involved in the carotenoid biosynthesis pathway in *D*. *salina* have been cloned. *β*-carotene production in *D*. *salina* is enhanced by suboptimal growth conditions, such as high irradiance, high salinity, low temperature, nutrient deprivation and heavy metal stress [11]. Transcriptional regulation is likely to be an important step of carotenoid biosynthesis pathway control in *D*. *salina* [12]. However, contradictory information is available concerning the transcriptional regulation of the carotenoid biosynthesis pathway in *D*. *salina*. For example, the regulatory roles of *PSY* and *PDS* are unclear because many studies of the transcriptional and translational regulation of *PSY* reached contradicting conclusions. Specifically, under carotenogenic conditions, no up-regulation of *PSY* (nitrogen deprivation) or *PDS* (high light) was observed at the transcriptional or translational level [8, 13], but another study of the same species observed increased gene expression for both genes [14]. A mechanism that is independent of the direct regulation of the carotenoid biosynthesis pathway has also been suggested, in which the production of *β*-carotene-sequestering structures (i.e., lipid globules) increases in response to environmental stress, thereby creating a plastid-localized sink for *β*-carotene [8]. Based on the finding that no transcriptional and translational upregulation of *PSY* and *PDS* occurred under carotenogenic conditions, the authors further hypothesized that the carotenoid biosynthesis pathway enzymes are often not maximally active under non-inducing conditions and that the *β*-carotene sink might stimulate their activity by sequestering *β*-carotene, thus avoiding end-product inhibition of the carotenoid biosynthesis pathway [8]. Therefore, the molecular mechanism that regulates the carotenoid biosynthesis pathway remains unclear.

Nitrogen, sulfur and phosphorus are essential plant macronutrients [15]. Nitrate availability has been proved to be a fundamental factor that influences cell growth and carotenoid accumulation in *D*. *salina* [12, 14, 16, 17, 18, 19]. The effects of sulfur deprivation on metabolite partitioning, growth characteristics, pigment content, the rates of photosynthesis and respiration, and endogenous substrates (starch and protein) have been investigated [16, 20]. Only one report on phosphorous deprivation in *D*. *salina* has been published to date [16]. Therefore, systematic studies of the physiological and molecular responses of *D*. *salina* to macronutrient deprivation are indispensable for understanding the molecular basis of the carotenoid biosynthesis pathway. In the present study, the physiological and molecular responses to the deprivation of nitrogen (-N), sulfur (-S), phosphorous (-P) and different combinations of those nutrients (-N-P, -N-S, -P-S, -N-P-S) were investigated. Based on the carotenoid accumulation results and the transcriptional levels of carotenoid biosynthesis pathway genes observed during nutrient deprivation, we proposed that the regulation of the carotenoid biosynthesis pathway occurs at both the transcriptional and posttranscriptional levels.

## Materials and Methods

### Culturing

*D*. *salina* strain TG (isolated from Tanggu, China) was cultured in modified Johnson’s medium (S1 Table). We devised -N, -P, -S, -N-P, -N-S, -P-S, and -N-P-S nutrient limitation media and a complete modified Johnson’s medium (CM) for *D*. *salina* cultures. For these nutrient limitation media, equimolar KCl was used instead of KNO_3_, equimolar KCl was used instead of KH_2_PO_4_, and equimolar MgCl_2_ was used instead of MgSO_4_. The alga was grown at 30°C in 0.5-L Erlenmeyer flasks containing 250 mL of medium under continuous illumination (60 μmol photons m^-2^ s^-1^, fluorescent lamp, 400–700 nm). The cultures were shaken manually once each day. The cells were inoculated at 2.0× 10^5^ mL^-1^. Cells were cultured for two 16/8 hour light/dark cycles to synchronize the growth phases before inoculation and transfer to continuous light conditions. For the inoculation of nutrient deprivation experiments, the cells were prewashed three times with a 2 M NaCl solution to eliminate nutrient remnants from the medium. At least three experimental replicates were set for each measurement in this study.

### Cell Density Determination

Cell counts were performed every 2 days using a hemocytometer. The cells were fixed with glutaraldehyde (0.25% final concentration) for 2 min, then counted using an Olympus CX40 microscope (Olympus Corporation, Tokyo, Japan) and a hemocytometer. All data in this study were analyzed using Origin 9.0.0 (OriginLab Corporation, Northampton, MA). Analyses of statistical significance were performed using SPSS v19.0 (IBM, Armonk, NY). One-way ANOVA was used in this study, followed by the Least Significant Difference Test for post-hoc analysis.

### Chlorophyll and Total Carotenoid Analysis

Pigment extraction was carried out according to a previously described method [21]. A 5-mL aliquot of a *D*. *salina* culture was centrifuged at 4,000 rpm for 2 min. The pellet was washed with fresh medium, suspended in 5 mL of an 80% (v/v) acetone solution, thoroughly vortexed and extracted for one night in the dark until the pellets turned clear. The absorbance of the relevant pigments in the extract was measured using a UVmini-1240 (Shimadzu, Kyoto, Japan) spectrophotometer. The whole extraction procedure was carried out under dim light. Chlorophyll and total colored carotenoids were estimated according to a previously described method, as follows [22]: Chl *a* (μg mL^-1^) = 11.75(A_662_)-2.35(A_645_); Chl *b* (μg mL^-1^) = 18.61(A_645_)-3.96(A_662_)Cx+c(μg mL^-1^) = (1000 A_470_-2.270Chl*a*-81.4Chl*b*)/198, where Cx+c is the total colored carotenoids.

### Analysis of *β*-Carotene and Lutein by HPLC

Carotenoid extraction was carried out according to a previously described method [23]. A 5-mL aliquot of a *D*. *salina* culture was centrifuged at 2,500 rpm for 2 min. The pellet was washed with a 2 M sodium chloride solution, re-suspended with 5 mL of methanol/ methylene chloride (75/25, v/v), thoroughly vortexed and extracted at room temperature for 20 min until the pellets turned clear. Then, the solution was centrifuged at 13,000 rpm for 5 min and filtered through a 0.22-μm membrane filter for HPLC analysis. The whole extraction procedure was carried out under dim light. HPLC was performed using a Dionex P680 (Sunnyvale, CA). The analysis was carried out with a reverse-phase BDS HYPERSIL C18 (250×4.6 mm, 5.0 μm). The samples were eluted using acetonitrile/methylene chloride/methanol (85/10/5, v/v/v) as mobile phases. Elution was carried out at 1 mL min^-1^ using a 20-μL injection volume loop with a micro-syringe, with a detection wavelength and column temperature of 450 nm and 28°C, respectively. The identity of *β*-carotene and lutein was confirmed by comparing the HPLC retention times with those of the analytical standards at a wavelength of 450 nm. At least three experimental replicates were set for each measurement. *β*-carotene and lutein were purchased from Sigma-Aldrich and used for calibration. Concentrations (mg L^–1^) of *β*-carotene were determined using the following equation: $X\;=\;\frac{A+0.396}{2.184}$, Where X is the *β*-carotene content (mg L^-1^) and A is the area count. The correlation coefficient of the curve was 0.9995. The content of lutein was shown by the relative content per cell.

### Extraction and activity analysis of SOD and CAT

The crude extracts were prepared according to a previously described method [24], with some modifications. A 30-mL aliquot of a *D*. *salina* culture was centrifuged at 6,000 rpm for 10 min at room temperature. The pellets were weighed, transferred to a 1.5-mL centrifuge tube and resuspended with 1 mL of sodium phosphate buffer (50 mM, pH 7.8) and 0.02 g of polyvinylpyrrolidone (PVP). The suspension was centrifuged at 12,000 rpm for 20 min at 4°C to crumble the cells, and the supernatant was collected by centrifugation at 13,000 rpm for 30 min at 4°C. The supernatant was the enzyme extract and was used directly for enzyme activity analysis or diluted by 50% (w/v) with glycerol to maintain its activity at -20°C until the activity analysis. The superoxide dismutase (SOD) reaction mixture contained 1 mL of methionine (39 mM), 30 μl of enzyme extract, 1 mL of nitroblue tetrazolium (NBT, 189 μM) and 1 mL of riboflavin (6 μM). The mixtures were illuminated with a fluorescent lamp (60 μmol photons m^-2^ s^-1^) for 20 min at 30°C, and the absorbance was determined at 560 nm. The blank contained 1 mL of methionine (39 mM), 1 mL of NBT (189 μM), 30 μl of sodium phosphate buffer (50 mM, pH 7.8) and 1 mL of sodium phosphate buffer (50 mM, pH 7.8) and was kept in a dark place. A solution that contained 1 mL of methionine (39 mM), 30 μL of sodium phosphate buffer (50 mM, pH 7.8), 1 mL of NBT (189 μM), 30 μl of sodium phosphate buffer (50 mM, pH 7.8) and 1 mL of riboflavin (6 μM) served as the largest reduction tube. One unit (U) of SOD was defined as the amount of enzyme that caused a 50% decrease in the SOD-inhibitable NBT reduction. SOD activity was calculated using the following equation: SOD Activity (U g^-1^) = $\frac{(A_{c}-A_{s})\times V}{\frac{1}{2}\times A_{c}\times FW\times V_{t}}$, Where A_C_ is the absorbance of the control, A_S_ is the absorbance of the sample, V is the total enzyme extract volume (mL), V_t_ is the enzyme extract volume in the sample reaction (mL), and FW is the sample fresh weight (g). The activity of catalase (CAT) was determined as follows: the reaction mixture contained 2.9 mL of H_2_O_2_ (1 mM) and 0.1 mL of enzyme extract, and the blank contained 2.9 mL of H_2_O_2_ (1 mM) and 0.1 mL of sodium phosphate buffer (50 mM, pH 7.8). The change in absorbance during 40 s was determined at 240 nm. One CAT unit (U) was described as the amount of enzyme for which the A_240_ reduction was 0.01 in 1 min. CAT activity was calculated using the following equation: CAT activity (U/g) = $\frac{\Delta A_{240}\times V_{t}}{FW\times V\times 0.01\times t}$, Where Δ A_240_ is the change in the sample during 40 s, FW is the sample fresh weight (g), t is the reaction time, V is the total enzyme extract volume (mL), and V_t_ is the enzyme extract volume in the sample reaction (mL).

### Measurement of Photosynthetic Rates

The photosynthetic and respiration rates of *D*. *salina* were measured using an Oxylab Clark-type electrode (Hansatech, Cambridge, UK). A total of 2.5 mL of cell culture were added to the reaction chamber and treated with light and dark for 3 min each. Then, the changes in dissolved oxygen in the medium were measured to calculate the photosynthetic oxygen evolution and respiratory oxygen consumption. At least three experimental replicates were set for the measurement. The photosynthetic, respiration and real photosynthetic rates were calculated using the equations described below [25]. Y_1_(μmol · mgChl^−1^ · h^−1^) = $\frac{(C_{2}-C_{1})\times V}{Chl\times t}$, where Y_1_ is the net photosynthetic rate, *V* is the volume of the sample, Chl is the chlorophyll content in the samples, t is the reaction time, C_1_ is the initial concentration of oxygen, C_2_ is the concentration of oxygen after 3 min under light conditions. Y_2_(μmol · mgChl^−1^ · h^−1^) = $\frac{(C_{2}-C_{3})\times V}{Chl\times t}$, where Y_2_ is the respiration rate, V is the volume of the sample, Chl is the chlorophyll content in the samples, t is the reaction time, C_2_ is the concentration of oxygen after 3 min under light conditions, and C_3_ is the concentration of oxygen after 3 min under dark conditions. Y_3_ = Y_2_+Y_1_, Where Y_3_ is real photosynthesis.

### Microscopy

Bright field microscopy images of cells and globule suspensions were captured using an Olympus BX53F fluorescence microscope (Olympus Corporation, Tokyo, Japan) with an Olympus DP72 digital color camera (Olympus Corporation, Tokyo, Japan). For neutral lipid fluorescence observation, the cells were harvested by centrifugation at 2,500 rpm for 5 min and resuspended in fresh medium at 1×10^6^ cells per mL. Nile Red dissolved in acetone was added to a concentration of 100 μg mL^-1^. Chlorophyll (Fluorescence Mirror Unit U-MWU2, excitation 330 nm, emission 400 nm) and Nile-red fluorescence (Fluorescence Mirror Unit U-RFP, excitation 531 nm, emission 562 nm) images were acquired and processed using Image-Pro Express 6.3 (Glen Mills, PA). The stored solution was added to the sample (to a final concentration of 1μg mL^-1^).

### RNA Extraction and qRT-PCR

Cells subjected to each type of nutrient deprivation at each time point were harvested by centrifugation at 2,500 rpm for 5 min and then immediately stored in liquid nitrogen until RNA extraction. The cells were ground into a powder, and RNA was extracted and purified using plant-specific protocols for the QIAGEN RNeasy Mini Kit (Qiagen Inc., Valencia, CA). RNA concentrations and quality were determined using a BioSpectrometer^®^ basic (Eppendorf, Germany) and agarose gel electrophoresis. Total RNA was used to synthesize oligo (dT)_18_-primed cDNA with the RevertAid First Strand cDNA Synthesis Kit, according to the manufacturer’s instructions. qRT-PCR was performed on a StepOne^™^ Real-Time PCR System (Applied Biosystems, Foster City, CA) by using the DyNAmo Color Flash SYBR Green qPCR Kit (Thermo Fisher Scientific, Waltham, MA). All reactions were performed in triplicate. No-template controls were included in each PCR, and the data were normalized according to the 18S rRNA gene [14]. The following thermal profile was used for all PCR reactions: 95°C for 2 min, 40 cycles at 94°C for 15 s, 60°C for 1 min. Amplicon dissociation curves were obtained after cycle 40 by heating from 60°C to 95°C with a ramp speed of 0.3°C per min. The specificity of PCR amplification was confirmed by the presence of dissociation curves with single peaks and unique amplicons of the expected size upon electrophoresis on agarose gels. The data were analyzed using the StepOne Software v2.3 (Thermo Fisher Scientific, Waltham, MA). Gene expressions of cells cultured in CM for seven days were used as control. All quantifications were normalized to the amount of 18S rRNA as an internal standard [14] using the ΔΔC_T_ method. The sequences of the primers are listed in S2 Table. The coding sequences of these genes were retrieved from the NCBI nucleotide database (http://www.ncbi.nlm.nih.gov/nuccore).

## Results

### Retarded Growth and the Accumulation of Lipid Droplets during Nutrient Deprivation

**Fig 1:**
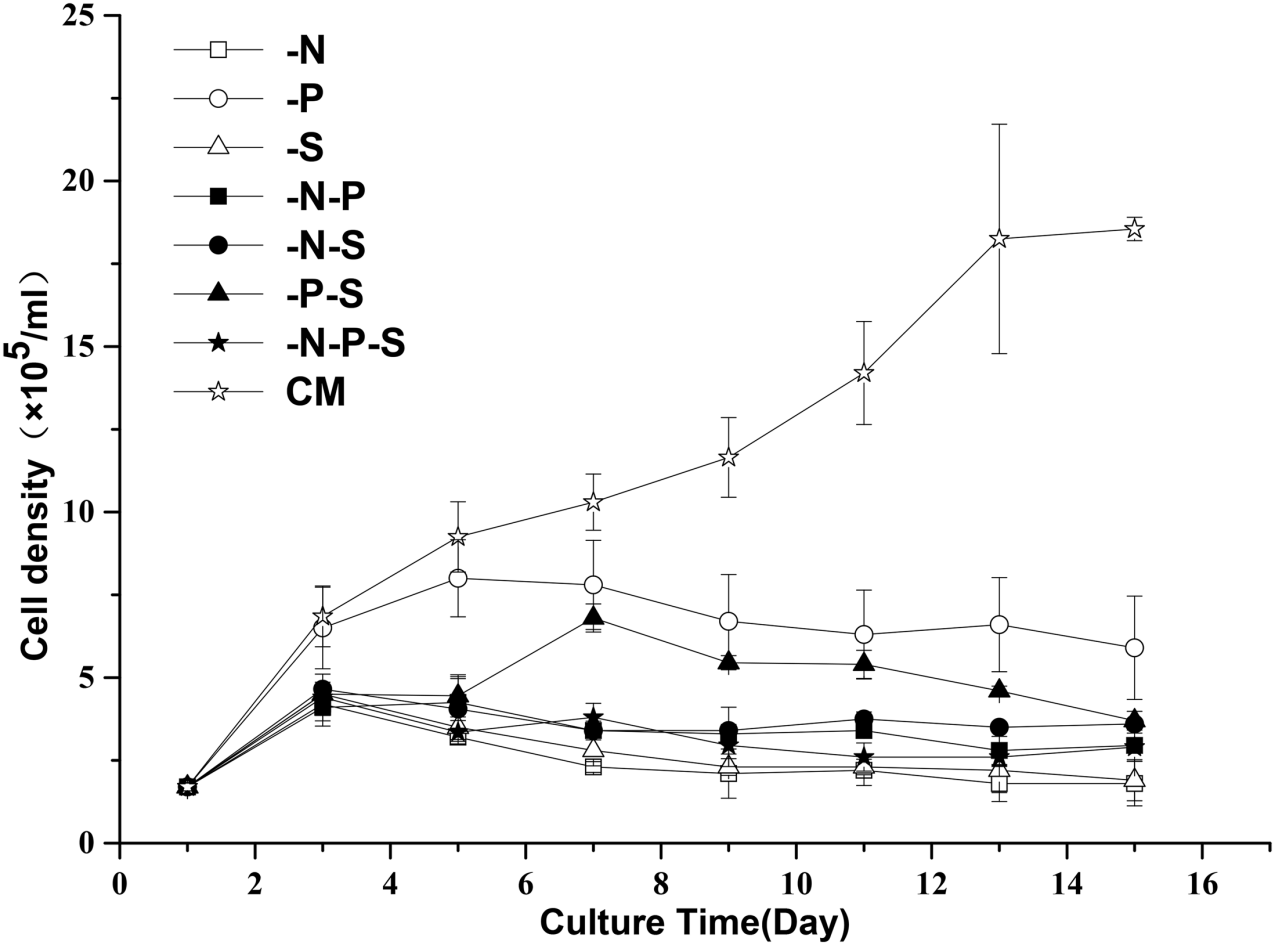
The effects of nutrient deprivation on the cell growth of *D*. *salina*. Cell density was determined for 15 days after exposure to the different treatments. Complete medium (CM), Nitrogen deprivation (-N), Phosphorous deprivation (-P), Sulfur deprivation (-S), Nitrogen and phosphorous deprivation (-N-P), Nitrogen and sulfur deprivation (-N-S), Phosphorous and sulfur deprivation (-P-S), Nitrogen, phosphorous and sulfur deprivation (-N-P-S). The plotted data are the averages ± SE of six replicates.

**Fig 2:**
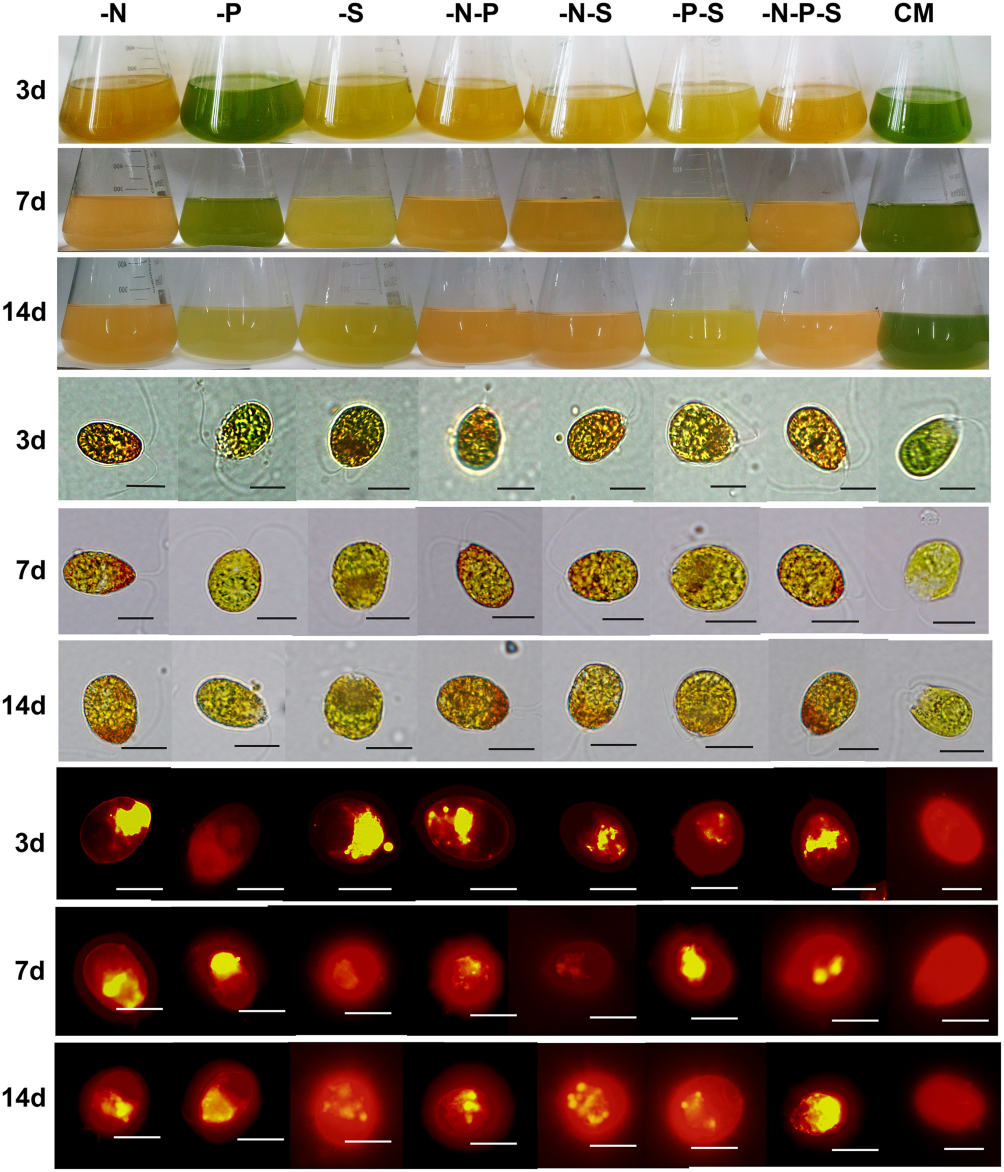
Changes in culture color and the morphology of *D*. *salina* cells. The first three rows of this picture demonstrate that the color of the cultures changed gradually during nutrient deprivation. The middle three rows of this picture demonstrate that the color of the cells changed gradually during nutrient deprivation. The last three rows of this picture show that the lipid globules changed during nutrient deprivation. Complete medium (CM), Nitrogen deprivation (-N), Phosphorous deprivation (-P), Sulfur deprivation (-S), Nitrogen and phosphorous deprivation (-N-P), Nitrogen and sulfur deprivation (-N-S), Phosphorous and sulfur deprivation (-P-S), Nitrogen, phosphorous and sulfur deprivation (-N-P-S). Scale bars, 10 μm.

Nutrient deprivation is a mild stress in comparison to high light intensity and high salt concentrations. Initially, we investigated the effects of nutrient deprivation on the growth and morphology of *D*. *salina* cells. The results showed that the growth of *D*. *salina* was suppressed when the cells were subjected to nutrient deprivation, but the inhibitory effects of the seven combinations of nutrient deprivation (-N, -P, -S, -N-P, -N-S, -P-S, -N-P-S) on the growth of *D*. *salina* were different (Fig 1). Depriving nitrogen (-N, -N-P, -N-S, -N-P-S) and sulfur (-S, -S-P) resulted in partial growth arrest before the 3rd day and complete growth arrest after the 3rd day. Depriving phosphorous alone (-P) resulted in partial growth arrest before the 5th day and generated a higher cell density than the other nutrient deprivation combinations. Interestingly, another growth peak was observed on the fifth day after combined sulfur and phosphorous deprivation (-P-S). The color of the cultures changed gradually during nutrient deprivation (Fig 2). Depriving nitrogen (-N, -N-P, -N-S, -N-P-S) resulted in faster changes in the color of the cultures and the cells (Fig 2) than those of depriving phosphorous and sulfur (-P, -S, -S-P). The color of the cultures and the cells changed slowly after phosphorous deprivation. As lipid globules are carotenoid-sequestering structures, these globules can be visualized by Nile Red staining (Fig 2). The results showed that lipid globules accumulated under nutrient deprivation conditions. It is worth noting that cells subjected to the single deprivation of phosphorous accumulated more lipid globules than cells subjected to the single deprivation of sulfur. In addition, the cells retained their flagella under all types of nutrient limitation conditions.

### Decreased Chlorophyll Contents and Photosynthetic Rates during Nutrient Deprivation

**Fig 3:**
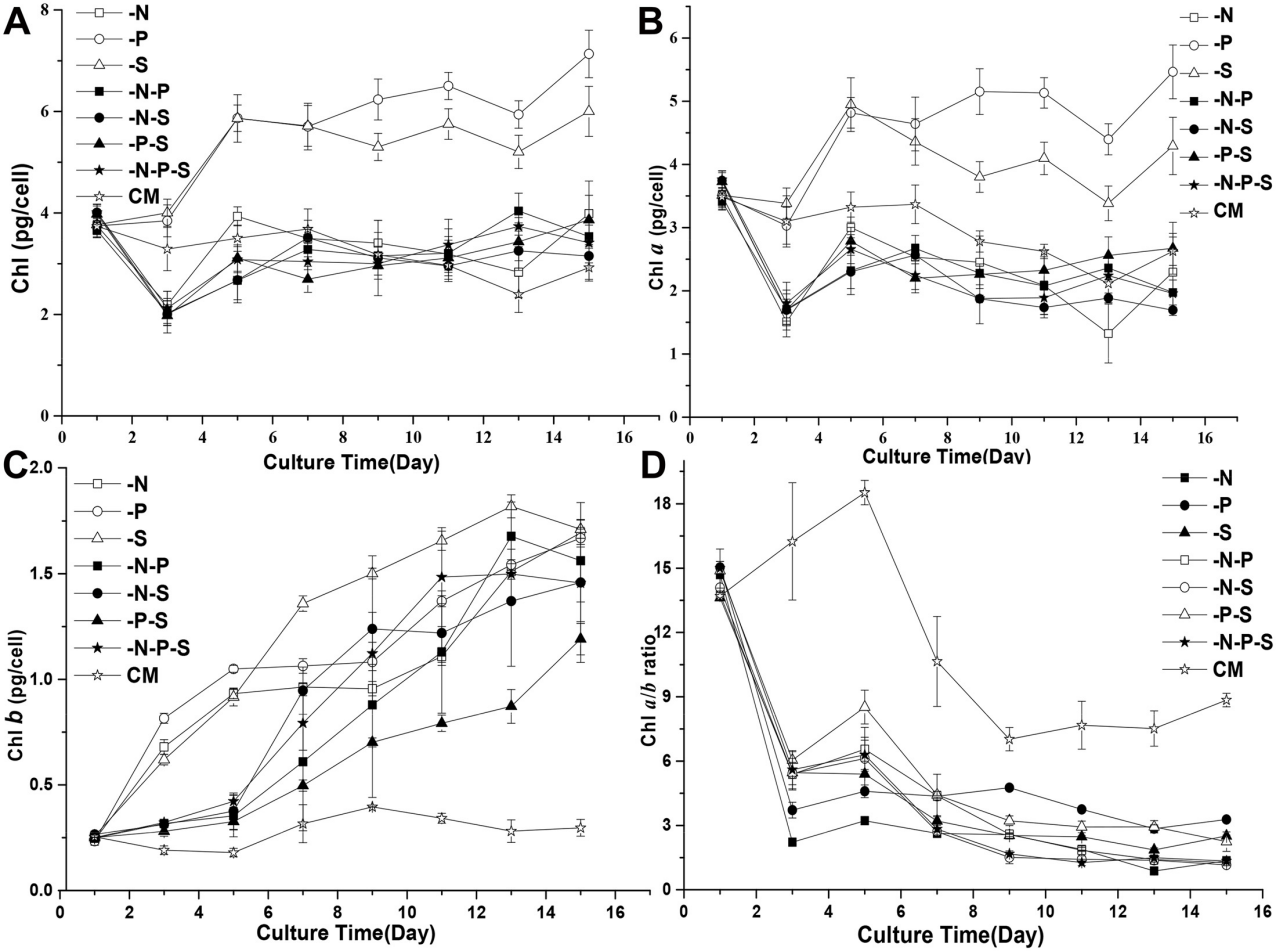
The chlorophyll contents of *D*. *salina* cells during nutrient deprivation. Changes in the total chlorophyll (A), chlorophyll a (B) and chlorophyll b (C) contents were measured during nutrient deprivation. The ratio of chlorophyll a and chlorophyll b (D) during nutrient deprivation was used to reflect changes in the status of the cells. Complete medium (CM), Nitrogen deprivation (-N), Phosphorous deprivation (-P), Sulfur deprivation (-S), Nitrogen and phosphorous deprivation (-N-P), Nitrogen and sulfur deprivation (-N-S), Phosphorous and sulfur deprivation (-P-S), Nitrogen, phosphorous and sulfur deprivation (-N-P-S). The presented data are the averages ± SE of three replicates.

**Fig 4:**
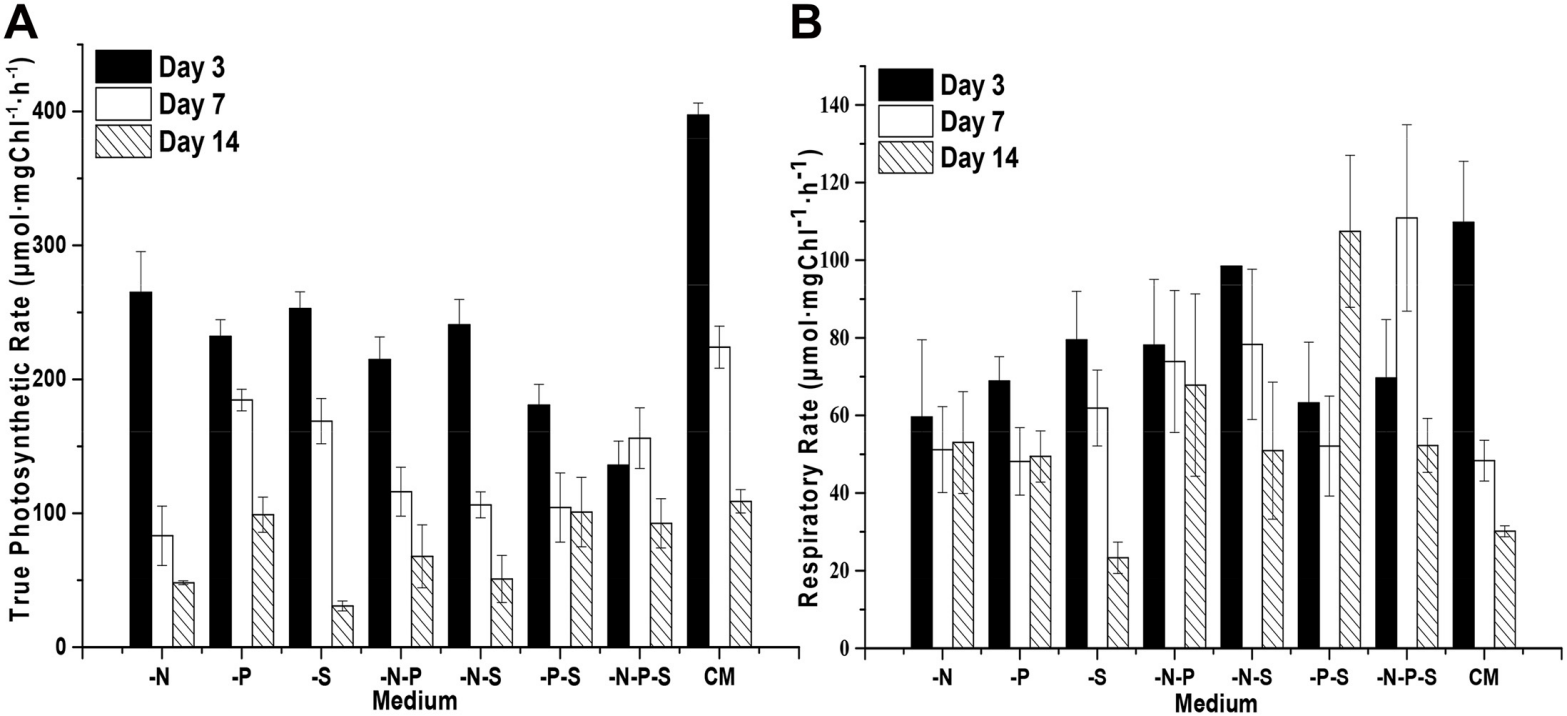
The photosynthetic and respiratory rates of *D*. *salina* cells during nutrient deprivation. The photosynthetic and respiratory rates were calculated based on the amount of oxygen consumed and released by *D*. *salina* cells under dark and light conditions, respectively. The true photosynthetic rates (A) show the changes in the amount of oxygen released, and the respiratory rates (B) show the changes in the amount of oxygen consumed. Complete medium (CM), Nitrogen deprivation (-N), Phosphorous deprivation (-P), Sulfur deprivation (-S), Nitrogen and phosphorous deprivation (-N-P), Nitrogen and sulfur deprivation (-N-S), Phosphorous and sulfur deprivation (-P-S), Nitrogen, phosphorous and sulfur deprivation (-N-P-S). The presented data are the averages ± SE of three replicates.

Considering the changes in the color of the cultures and cells during nutrient deprivation, the contents of the photosynthetic pigments chlorophyll *a* (Chl *a*) and *b* (Chl *b*) were analyzed. The results showed that the contents of Chl (Chl *a* and *b*) changed upon nutrient deprivation. The contents of Chl increased and reached a plateau after five days under the -P and -S conditions (Fig 3A). Under the -N, -N-S, -N-P, -N-S and -N-P-S conditions, the Chl contents decreased before the 3rd day and increased to the initial content of the cells after several more days. The contents of Chl *a* exhibited a trend similar to that of total Chl during nutrient deprivation (Fig 3B). However, the contents of Chl *b* increased during combined nutrient deprivation (Fig 3C). Therefore, the Chl *a/b* ratios decreased during combined nutrient deprivation (Fig 3D). For the *D*. *salina* cells cultured in complete medium (Johnson’s medium) [26], the Chl *a/b* ratio increased before the fifth day and decreased afterward. The photosynthetic rates were calculated from the amount of oxygen consumed and released by *D*. *salina* cells in the dark and in the light, respectively. The results showed that the true photosynthetic rates of *D*. *salina* cells decreased during nutrient deprivation and gradually decreased with additional culturing time both during nutrient deprivation and in complete medium (CM) (Fig 4A). It is worth noting that the true photosynthetic rates of the *D*. *salina* cells decreased most significantly under -N-P-S conditions, without further decrease before the 7th day, however, the true photosynthetic rates were higher than those observed under -N and -S conditions on the 15th day. Similar phenomena occurred under -N-P and -P-S conditions. The respiratory rates of *D*. *salina* cells decreased and changed insignificantly (P>0.05) with additional culturing time under -N and -N-P conditions in comparison to cells grown in CM (Fig 4B). For the -P, -S and -N-S conditions, the respiratory rates of *D*. *salina* cells decreased with extending culturing time. For the -P-S condition, the respiratory rates decreased before the 7th day and then increased to the initial level. Interestingly, during the combined deprivation of nitrogen, phosphorous and sulfur (-N-P-S), the respiratory rates decreased, followed by an increase and another subsequent decrease (Fig 4B).

### Accumulated Carotenoids during Nutrient Deprivation

**Fig 5:**
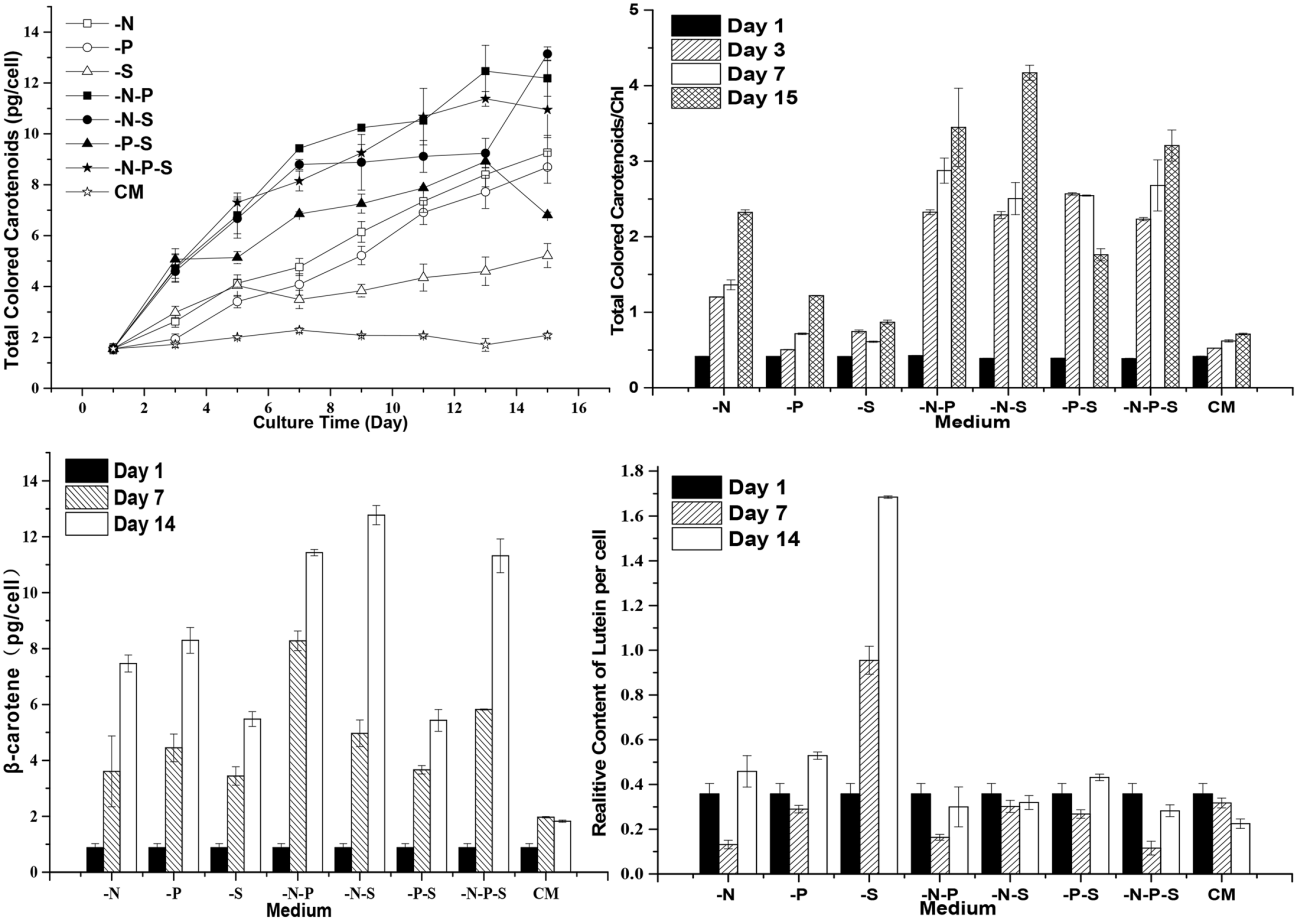
The carotenoid contents of *D*. *salina* cells during nutrient deprivation. The total carotenoid contents (A) were measured using the colorimetric method. The ratio of total colored carotenoids to chlorophyll (B) indirectly reflected the changes in carotenoids and chlorophyll in *D*. *salina* cells. The *β*-carotene contents (C) and the relative contents of lutein (D) were measured via HPLC. Complete medium (CM), Nitrogen deprivation (-N), Phosphorous deprivation (-P), Sulfur deprivation (-S), Nitrogen and phosphorous deprivation (-N-P), Nitrogen and sulfur deprivation (-N-S), Phosphorous and sulfur deprivation (-P-S), Nitrogen, phosphorous and sulfur deprivation (-N-P-S). The presented data are the averages ± SE of three replicates.

To investigate the effects of nutrient deprivation on the carotenoid metabolism pathway, the total contents of carotenoids, *β*-carotene and lutein were analyzed. Total colored carotenoids were increased upon nutrient deprivation. Surprisingly but not unexpectedly, many lipid droplets accumulated under -P conditions (Fig 2). The -P treatment resulted in an increase in accumulating carotenoids after the 3rd day and higher contents of total colored carotenoids after the 7th day in comparison to the single deprivation of sulfur (Fig 5A). In addition, the *D*. *salina* cells looked greener under -P conditions than under -S conditions. The total colored carotenoids contents were higher during the deprivation of multiple nutrients than during the deprivation of a single nutrient (i.e., nutrient deprivation has cumulative effects on the total carotenoid accumulation when comparing the deprivation of multiple and single nutrients). During the deprivation of phosphorous and sulfur, the contents of total colored carotenoids decreased after the thirteenth day and were lower than those observed under -N and -P conditions. It is worth noting that no cumulative effect was observed between the deprivation of triple and double nutrients (P>0.05), and the -N-P and -N-S conditions even resulted in higher contents of total colored carotenoids than the -N-P-S condition on the fifteenth day. Accordingly, the ratios of total colored carotenoids/Chl were increased under nutrient deprivation conditions (Fig 5B). Lycopene can be cyclized into *β*-carotene or *α*-carotene in *D*. *salina*, as mentioned above. *α*-carotene can be hydrolyzed into lutein. In *D*. *salina*, in addition to *β*-carotene, lutein is another important carotenoid that protects cellular components from damage incurred by reactive oxygen species under stressful conditions [27, 28, 29]. The *β*-carotene contents of *D*. *salina* cells increased upon nutrient deprivation (Fig 5C). The -N-P and -N-S conditions also showed cumulative effects on *β*-carotene accumulation, in contrast to the -N, -P and -S conditions. As an exception, the -P-S condition resulted in *β*-carotene contents (P>0.05) similar to those observed for the–S condition, but the *β*-carotene contents under the -P-S condition were smaller than those observed under the -P condition. Similarly, no cumulative effect was observed between the deprivation of triple and double nutrients (P>0.05). There were no coincident changes in the contents of lutein during nutrient deprivation (Fig 5D). The lutein contents of the cells under -N, -P and -N-P conditions decreased before the 7th day and then increased. The lutein contents under the -S condition increased with extending culturing time. Interestingly, the -N-S, -P-S and -N-P-S conditions resulted in no significant changes in the lutein contents of *D*. *salina* cells. For the cells cultured in CM, the lutein contents decreased gradually with extending culturing time.

### Altered Antioxidant Enzyme Activities during Nutrient Deprivation

**Fig 6:**
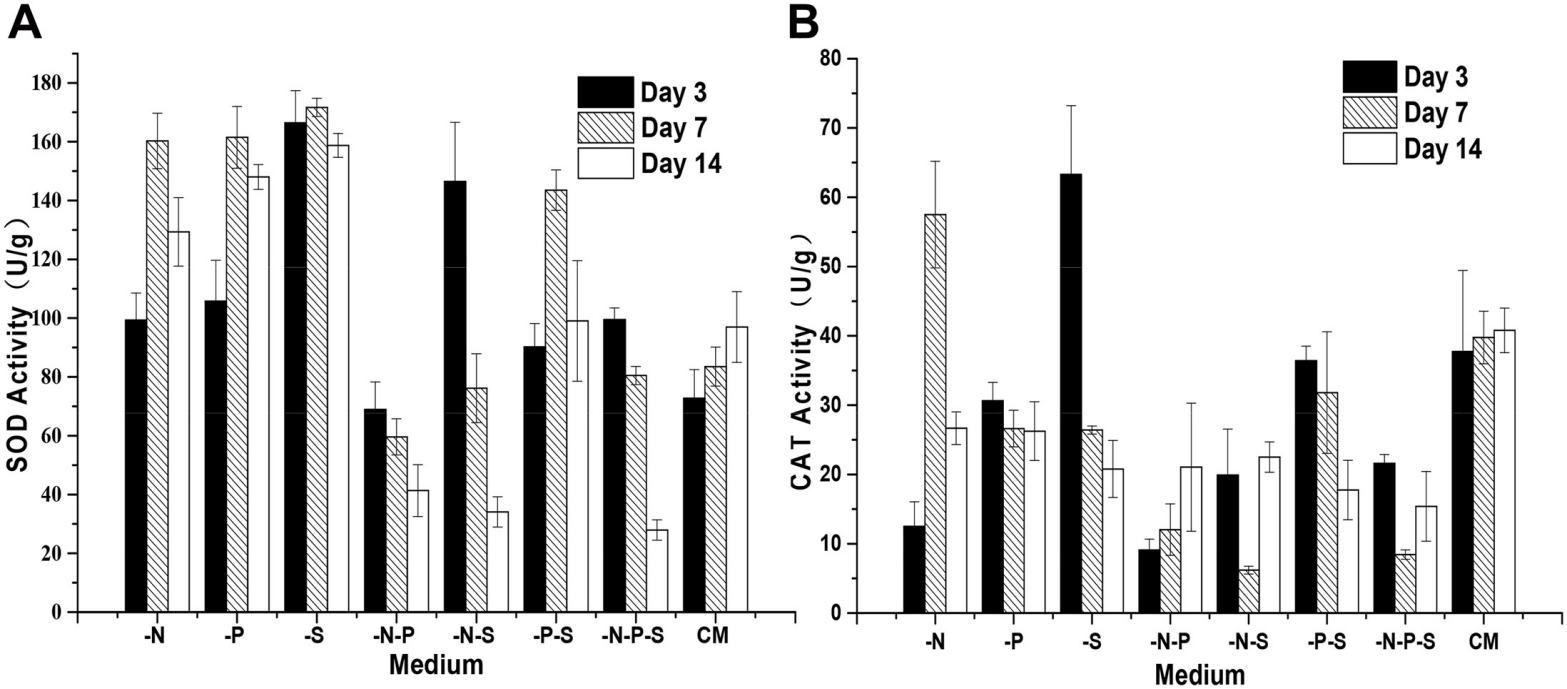
The activities of the antioxidant enzymes of *D*. *salina* during nutrient deprivation. The activities of SOD (A) and CAT (B) in *D*. *salina* cells during nutrient deprivation were determined using a colorimetric method. Complete medium (CM), Nitrogen deprivation (-N), Phosphorous deprivation (-P), Sulfur deprivation (-S), Nitrogen and phosphorous deprivation (-N-P), Nitrogen and sulfur deprivation (-N-S), Phosphorous and sulfur deprivation (-P-S), Nitrogen, phosphorous and sulfur deprivation (-N-P-S). The presented data are the averages ± SE of three replicates.

Like other photosynthetic organisms, algal cells have a high internal oxygen concentration due to oxygenic photosynthesis. Reactive oxygen species (ROS) activated by oxygen constantly threaten photosynthetic organisms [30]. It has been suggested that ROS are involved in triggering massive *β*-carotene accumulation in *D*. *salina* under stress conditions [31]. The accumulation of *β*-carotene induced by ROS-generating herbicides is accompanied by increased activities of antioxidant enzymes, such as SOD and CAT [32]. Therefore, we investigated the activities of SOD and CAT in *D*. *salina* cells during nutrient deprivation. Results showed that the activities of SOD were increased under -N, -P and -S conditions in comparison to cells grown in CM (Fig 6A). For the cells grown under -N, -P and -S conditions, the activities of SOD increased initially and then decreased. The observed changes in the activities of SOD under the -P-S condition were similar to those observed during single nutrient deprivation. The activities of SOD under -N-P, -N-S and -N-P-S conditions decreased gradually with extending culturing time. However, the activities of SOD under -N-P conditions were lower than those observed for cells grown in CM and decreased gradually during the nutrient deprivation process. The activities of CAT remained constant (P>0.05) during 14 days of cultivation in CM (Fig 6B). Under -N conditions, the activities of CAT were decreased by 66.8% in comparison to cells grown in CM on the 3rd day. With extending culturing time, the activities of CAT were increased by 44.6% on the 7th day and decreased by 34.6% on the 14th day compared with cells grown in CM. Under -P conditions, the activities of CAT were decreased by 18.8%, and no significant differences (P>0.05) in comparison to cells grown in CM were observed among the three time points. Under -S conditions, the activities of CAT were increased by 67.8% on the 3rd day and then decreased by 33.6% and 49.1% on the 7th day and the 14th day, respectively, compared to cells grown in CM. The activities of CAT generally decreased during double and multiple nutrient deprivation. The activities of CAT under -N-P conditions decreased sharply and then increased gradually with the extension of the culturing time. For cells grown under -P-S conditions, no significant difference (P>0.05) were observed on the 3rd and 7th days, but a reduction by 55.5% occurred on the 14th day in comparison to cells grown in CM. Under -N-S and -N-P-S conditions, the activities of CAT decreased, with the lowest value on the 7th day.

### Altered Transcription of Carotenoid Biosynthesis pathway Genes during Nutrient Deprivation

**Fig 7:**
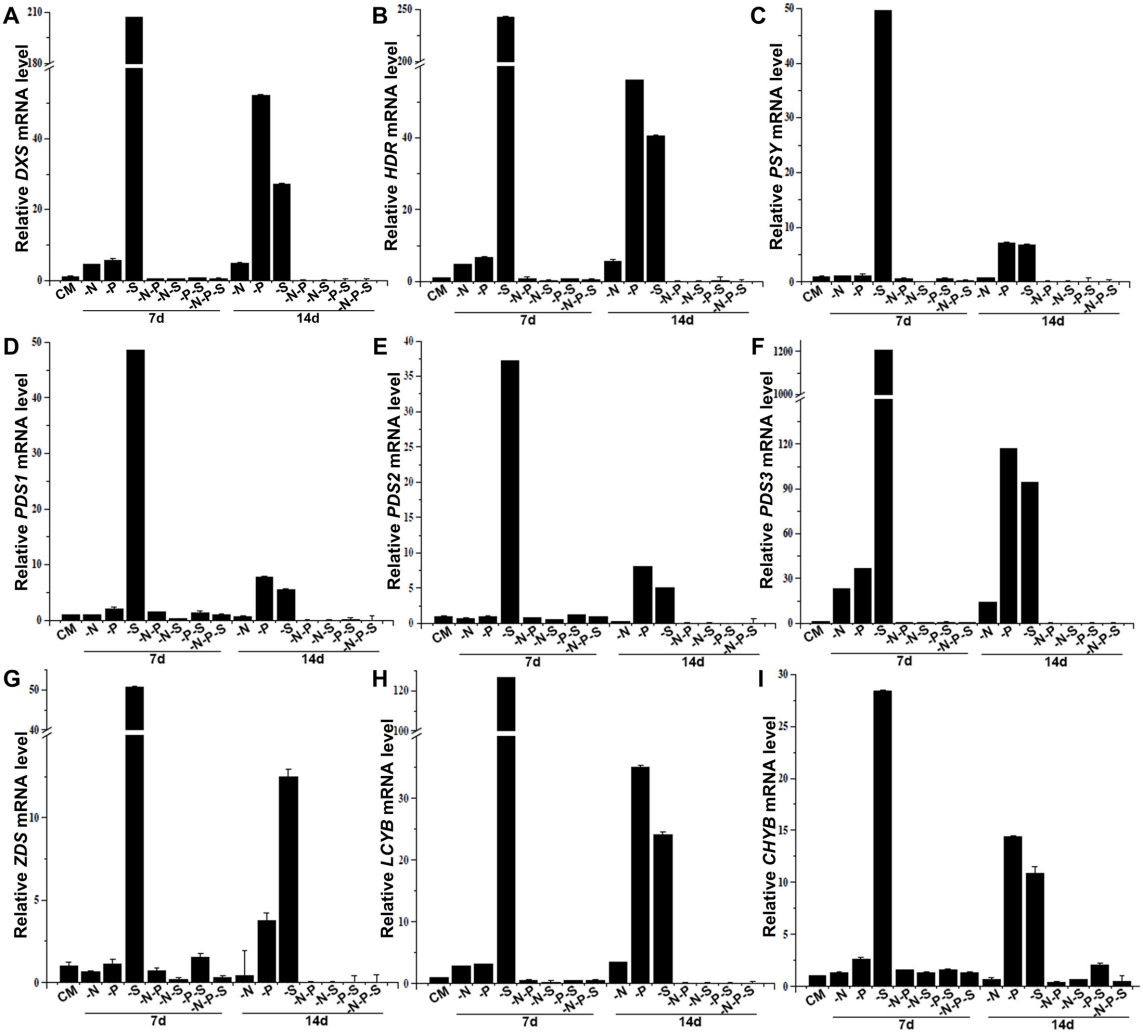
Steady-state mRNA levels of MEP and carotenoid biosynthesis pathway genes in *D*. *salina*. The cultures were subjected to different types of nutrient deprivation. As negative controls for nutrient deprivation, cultures were maintained in complete medium (CM). The samples used for RNA isolation were harvested 7 days and 14 days after exposure to the different treatments. The steady-state mRNA levels of *DXS* (A), *HDR* (B), *PSY* (C), *PDS1* (D), *PDS2* (E), *PDS3* (F), *ZDS* (G), *LCYB* (H) and *CHYB* (I) were quantified via quantitative real-time PCR, normalized against 18S rRNA, and plotted as the relative transcript levels versus time. Gene expressions of cells cultured in CM for seven days were used as control. Nitrogen deprivation (-N), Phosphorous deprivation (-P), Sulfur deprivation (-S), Nitrogen and phosphorous deprivation (-N-P), Nitrogen and sulfur deprivation (-N-S), Phosphorous and sulfur deprivation (-P-S), Nitrogen, phosphorous and sulfur deprivation (-N-P-S). The presented data are the averages ± SE of three replicates.

To investigate the effects of nutrient deprivation on the transcription of carotenoid biosynthesis pathway genes in *D*. *salina*, we retrieved the sequences for MEP and carotenoid biosynthesis pathway genes in *D*. *salina* and *D*. *bardawil* from the NCBI nucleotides database. The results showed that eleven different transcripts encoding MEP and carotenoid biosynthesis pathway enzymes have been deposited in the NCBI nucleotides database, including three transcripts for *P*SY (accession no: AY601075, EU328287, and DBU91900), three transcripts for *PDS* (accession no: GQ923693, AY954517 and Y14807), and single transcript for *DXS* (accession no: FJ469276), *ZDS* (accession no: HM754265), *HDR* (accession no: JQ762450), *LCYB* (Lycopene *β*-cyclase, accession no: EU327876), and *CHYB* (*β*-carotene hydroxylase, accession no: JN118489). PCR analysis indicated that the transcripts AY601075 and EU328287 were absent in both the genome and the transcriptome of the *D*. *salina* strain used in this study. PCR analysis and sequencing results indicated that all three transcripts of *PDS* (DQ845248.1, AY954517 and Y14807) are present in both the genome and the transcriptome of the *D*. *salina* strain used in this study. These results suggested that the evolutionary pergence of *PSY* and gene duplications of *PDS* occurred in different *D*. *salina* strains. The transcript levels of the nine genes were analyzed under different nutrient deprivation conditions using real-time RT-PCR, and the transcript levels of each gene under the CM condition were set as the control (Fig 7). Taking the *β*-carotene accumulation process under nutrient deprivation conditions into consideration, the 7- and 14-day time points were selected in the present study. The transcript levels of the nine genes were constant (P>0.05) in nutrient-sufficient CM (data not shown). The transcriptional patterns of the MEP pathway genes *DXS* and *HDR* were similar during nutrient deprivation (Fig 7A and 7B). The transcript levels of *DXS* and *HDR* under -N conditions were increased by 4.4 and 4.9 times, respectively, on the 7th day and by 4.9 and 5.6 times, respectively, on the 14th day. The transcript levels of *DXS* and *HDR* under -P conditions were increased by 5.7 and 6.6 times, respectively, on the 7th day and by 52.4 and 56.1 times, respectively, on the 14th day. Under -S conditions, the transcript levels of *DXS* and *HDR* were increased by 207.0 and 242.9 times, respectively, on the 7th day and by 27.3 and 40.6 times, respectively, on the 14th day. Under -N-P conditions, the transcript level of *HDR* showed no changes on the 7th day, but the transcript level of *DXS* was decreased by 2.1 times on the 7th the day. In addition, the transcript levels of *DXS* and *HDR* were further decreased by 121.0 and 150.0 times, respectively, on the 14th day. Under -N-S, -P-S and -N-P-S conditions, the transcript levels of *DXS* were decreased by 2.9, 1.6 and 2.0 times, respectively, on the 7th day and were further decreased by 92.0, 31.7 and 98.6, respectively, on the 14th day. Similarly, the transcript levels of *HDR* were decreased by 3.4, 1.5 and 2.0 times, respectively, on the 7th day and further decreased by 108.0, 13.7 and 93.8 times on the 14th day. Under -N conditions, the transcript level of *PSY* showed no significant changes compared with cells grown in CM, and no significant difference (P>0.05) was observed between the 7th and 14th days (Fig 7C). Under -P conditions, the transcript level of *PSY* was increased by 7.2 times on the 7th day. Under -S conditions, the transcript level of *PSY* was increased by 49.6 and 6.8 times on the 7th and 14th days, respectively. The transcript levels of *PSY* under -N-P, -N-S, -P-S and -N-P-S conditions were decreased by 1.4, 9.9, 1.9 and 3.0 times, respectively, on the 7th day and further decreased by 22.2, 15.5, 16.3 and 37.2 times, respectively, on the 14th day. The transcript level of *PDS1* (accession no: GQ923693) under -N conditions showed no changes on the 7th day but was decreased slightly by 1.7 times on the 14th day (Fig 7D). The transcript levels of *PDS2* (accession no: AY954517) on the 7th and 14th days under -N conditions were decreased by 1.5 and 3.1 times, respectively (Fig 7E). In contrast, the transcript levels of *PDS3* (accession no: Y14807) on the 7th and 14th days under -N conditions were increased by 23.2 and 14.2 times, respectively. The transcript levels of *PDS1* on the 7th and 14th days under -P conditions were increased by 2.1 and 7.8 times, respectively, and the transcript level of *PDS2* under -P conditions showed no changes on the 7th day but was increased by 8.0 times on the 14th day. The transcript levels of *PDS3* under -P conditions were increased by 36.8 and 117.4 times on the 7th and 14th days, respectively (Fig 7F). Under -S conditions, the transcript levels of *PDS1*, *PDS2* and *PDS3* were all increased sharply, by 48.7, 37.2 and 1207.5 times, respectively, on the 7th day and by 5.6, 5.0 and 94.5 times, respectively, on the 14th day. The transcript levels of *PDS1* and *PDS2* were decreased by 3.2 and 2.1 times, respectively, on the 7th day under -N-S conditions and showed no changes on the 7th day during other types of double and triple nutrient deprivation. In contrast, the transcript levels of *PDS1* were decreased by 35.1, 37.0, 10 and 84.8 times on the 14th day under -N-P, -N-S, -P-S and -N-P-S conditions, respectively. Similarly, the transcript levels of *PDS2* were decreased by 143.1, 84.1, 30.7 and 90.4 times on the 14th day under -N-P, -N-S, -P-S and -N-P-S conditions, respectively. The transcript levels of *ZDS* under -N conditions were decreased by 1.5 and 2.3 times on the 7th and 14th days, respectively (Fig 7G). The transcript levels of *ZDS* under -P conditions showed no changes on the 7th day but were increased by 3.8 times on the 14th day. In contrast to the changes observed under -N and -P conditions, the transcript levels of *ZDS* under -S conditions were increased by 50.8 and 12.5 times on the 7th and 14th days, respectively. The transcript levels of *ZDS* under -N-P, -N-S and -N-P-S conditions were decreased by 1.4, 5.0 and 3.0 times, respectively, without significant differences under -P-S conditions on the 7th day. The transcript levels of *ZDS* on the 14th day under -N-P, -N-S, -P-S and -N-P-S conditions were decreased by 625.0, 146.0, 68.0 and 346.0 times, respectively. The transcript levels of *LCYB* under -N, -P and -S conditions were increased by 2.8, 3.1 and 126.6 times, respectively, on the 7th day and by 3.4, 35.0 and 24.0 times, respectively, on the 14th day (Fig 7H). In contrast, the transcript levels of *LCYB* under -N-P, -N-S, -P-S and -N-P-S conditions were decreased by 1.9, 4.2, 2.1 and 2.3 times, respectively, on the 7th day and by 66.7, 51.0, 18.0 and 55.0 times, respectively, on the 14th day. The transcript levels of *CHYB* under -N conditions were slightly increased by 1.3 times on the 7th day and decreased by 1.6 times on the 14th day. The transcript levels of *CHYB* under -P and -S conditions were increased by 2.6 and 28.4 times, respectively, on the 7th day and by 14.4 and 10.8 times, respectively, on the 14th day (Fig 7I). In contrast, the transcript levels of *CHYB* on the 7th day under -N-P, -N-S, -P-S and -N-P-S conditions were increased by 1.6, 1.3, 1.6 and 1.3 times, respectively. On the 14th day, the transcript levels of *CHYB* under -N-P, -N-S and -N-P-S conditions were decreased by 2.6, 1.6 and 2.3 times, respectively, but the transcript levels of *CHYB* under -P-S conditions were increased by 2.0 times.

## Discussion

The most important finding of this study is that nutrient deprivation has cumulative effects on the total carotenoid content but not on the transcription of carotenoid biosynthesis pathway genes. Macronutrients play important roles in different aspects of plant development. Nitrogen and phosphorus are the basic component of biological macromolecules, such as proteins, DNA, RNA, ATP and the phospholipids that make up biomembranes, in plants. Sulfur is found in its reduced form in amino acids, peptides and proteins, in iron–sulfur clusters, and in lipoic acid and other co-factors and in its oxidized form as sulfonate group-modifying polysaccharides, proteins and lipids. Consistent with these facts, the growths of *D*. *salina* cells under -N and -S conditions decreased immediately and stopped after the 3rd day. Many phosphoesters play an essential role in metabolic reactions, particularly those that involve energy transfer. Phosphate is also a key component of signal transduction cascades establishing adaptive patterns of gene expression by protein phosphorylation and dephosphorylation [33]. Maintaining stable cytoplasmic phosphate concentrations is indispensable for many enzyme reactions [34]. The growth arrest of cells occurred on the fifth day under -P conditions. This result suggested that intracellular pools of phosphorous are present in *D*. *salina* cells, similar to higher plants. Salguero and colleagues [16] reported that no decrease in growth or increase in carotenoid contents occurred when the cells were subjected to phosphorous deprivation. Unfortunately, the sampling times were not given in that study.

Light energy is absorbed by chlorophyll or carotenoids, and the excited energy is transferred to the chlorophyll in the core complex of the photosystem. In parallel with the decreases in cell growth that occurred under nutrient deprivation conditions, the decreases in the true photosynthetic rates of *D*. *salina* cells under nutrient deprivation conditions suggested that the efficiency of the photosynthetic apparatus of *D*. *salina* cells was altered by nutrient deprivation. The observed changes in the contents of Chl *a* and *b* and the ratios of Chl *a*/*b* suggested that the structures of the photosynthetic apparatus were altered when the cells were subjected to nutrient deprivation. Previous studies showed the loss of PSII reaction center proteins in *D*. *tertiolecta* during continuous nitrogen limitation in chemostats [35]. The decreased levels of Chl *a* observed under nutrient deprivation conditions may also implicate similar processes in *D*. *salina*. Plants normally use Chl *a* as a pigment to oxidize water [36]. Consistently, both photosynthetic rates and Chl *a* are decreased under nutrient deprivation conditions. Chl *b* plays crucial roles in the accumulation of the light-harvesting chlorophyll a/b binding protein complex of photosystem II (LHCII). Interestingly, Chl *b* increased under all seven nutrient deprivation conditions. These results implied that more LHCII is needed to harvest light due to the accumulation of lipid globules with carotenoids.

The generation of ROS is one of the earliest responses of plant cells to various abiotic and biotic stresses. Generally, the antioxidant defense system of plant cells includes enzymatic and non-enzymatic antioxidants. CAT and SOD are the most efficient antioxidant enzymes. The combined action of those enzymes converts the potentially dangerous superoxide radical and hydrogen peroxide to oxygen and water, thus averting cellular damage. Increased activities of SOD under single nutrient deprivation conditions indicated that oxidative stresses occurred in *D*. *salina* cells subjected to nutrient starvation. On the other hand, the gradually decreasing activities of SOD under double or triple nutrient deprivation conditions indicated that the maintenance of SOD activity requires the presence of at least two of the three types of macronutrients. Based on our results, CAT may not play an essential role in ROS scavenging after nutrient starvation, although the activities of CAT were increased on the 3rd and 7th days under -S and -P conditions, respectively. It has been reported that the treatment of *D*. *salina* cells with the ROS-generating herbicide paraquat resulted in increased activities of SOD and CAT and the accumulation of *β*-carotene [32]. The carotenoids were sequestered primarily in lipid globules, according to previous reports [8] and our present study, but the photosystem on the thylakoid is the main site of ROS generation. Therefore, whether carotenoids, including *β*-carotene, serve as ROS-scavenging molecules remains an open question, and further study is needed in the future. As mentioned above, ROS are involved in triggering massive *β*-carotene accumulation in *D*. *salina* when the cells are exposed to stressful conditions [31], but the accumulated *β*-carotene is likely to be a consequence of the ROS generated under stress conditions and to serve as a parasol through which the photosystems avoid photoinhibition.

Earlier observations showed that transcription inhibitors prevented the massive accumulation of *β*-carotene in response to high light conditions in *D*. *salina* cells grown at high salinity, strongly suggesting that gene expression of carotenoid biosynthesis pathway enzymes may play an important role in the regulation of stress-induced carotenogenesis in this alga [37]. A previous study reported no up-regulation of *PSY* or *PDS* at the translational level during the overproduction of *β*-carotene induced by high light [8]. Taken together, we can conclude that the regulation of the carotenoid biosynthesis pathway occurs at the transcriptional and enzyme activity levels.

Consistent with our results, Sanchez-Estudillo and colleagues [13] reported that the transcript levels of *PSY* were constant under both nitrogen-sufficient and nitrogen-limited conditions based on a hetero-riboprobes ribonuclease protection assay. Our results clearly showed that the physiological and molecular responses of *D*. *salina* cells to various types of macronutrient deprivation are different. The transcript levels of *PSY* increased only under -S and -P conditions in our study. The results reported by Coesel and colleagues [14] indicated that the transcript levels of both *PSY* and *PDS* (identical to *PDS3* in the present study) were increased under nutrient deprivation conditions, as indicated by qRT-PCR. Because nutrient starvation was performed by diluting the medium with distilled water and correcting the salinity with NaCl without dissecting the regulatory effects of independent macro- and micronutrients, as done in this study, the results of the two studies cannot be compared. However, the increased levels of *PDS3* observed in this study and the increased levels of *LCYB* observed in another study that used the same procedure to generate nutrient starvation [18] are consistent with our results because these two genes were increased during the deprivation of all three kinds of macronutrients. As stated above, Rabbani and colleagues reported that the transcript level remained constant when the cells were subjected to high light stress, but unfortunately, the detailed sequence of the *PDS* gene used in that study was not provided. The transcriptional patterns of the three *PDS* genes during nutrient deprivation indicated that those genes play different roles in the regulation of the carotenoid biosynthesis pathway. The transcriptional up-regulation of *DXS* and *HDR* under three types of nutrient deprivation conditions suggested that the MEP pathway in *D*. *salina* was triggered to provide more substrate for carotenoid biosynthesis. A previous study reported that the transcriptional down-regulation of *DXS* occurs under nitrogen limitation conditions, based on a hetero-riboprobes ribonuclease protection assay [13]. The inconsistency between the two studies may suggest that there is not only one copy of *DXS* in the genome of *D*. *salina*. This discrepancy awaits the future release of the full genome of *D*. *salina*. The observed increase in the transcript levels of *CHYB* suggested that the transformation of *β*-carotene into other carotenoids was also activated under nutrient deprivation conditions. Moreover, the occurrence of the highest transcripts levels of MEP and carotenoid biosynthesis pathway genes under -S conditions suggested that sulfur plays a more important role in posttranscriptional regulation or enzyme activity levels than nitrogen and phosphorous. This process might involve a feedback mechanism to maintain the efficiency of MEP and the carotenoid biosynthesis pathway when more transcripts are needed for use as templates for protein translation. Another interesting finding is that the *D*. *salina* cells subjected to -S conditions accumulated more lutein than the cells grown under -N and -P conditions, which indicates that the regulation of lycopene *ε*-cyclase and lycopene *β*-cyclase was influenced differently by sulfur availability. Compared with the -N and -P conditions, the transcription of carotenoid biosynthesis pathway genes under -S conditions showed the greatest increase, but the total carotenoid and *β*-carotene contents under -S conditions were smaller than those observed under -N and -P conditions. These results suggested that the amounts or activities of carotenoid biosynthesis pathway enzymes under -N and -P conditions are higher than those that occur under -S conditions. These results suggested that the regulation of the carotenoid biosynthesis pathway in *D*. *salina* occurs at both the transcriptional and posttranscriptional levels (translation or enzyme activity).

## Supporting Information

S1 Table Components of culture medium. (DOCX)

S2 Table Primers used in qRT-PCR. (DOCX)

## Abbreviations

-N: Nitrogen deprivation
-S: Sulfur deprivation
-P: Phosphorus deprivation
-N-P: Nitrogen and phosphorus deprivation
-N-S: Nitrogen and sulfur deprivation
-P-S: Phosphorus and sulfur deprivation
-N-P-S: Nitrogen, sulfur and phosphorus deprivation
SOD: Superoxide dismutase
CAT: Catalase
MEP: Methylerythritol 4-phosphate
CM: Complete modified Johnson’s medium

## References

[1] Hunter WN. The non-mevalonate pathway of isoprenoid precursor biosynthesis. J Biol Chem. 2007; 282: 21573–21577. 17442674 10.1074/jbc.R700005200

[2] Eisenreich W, Bacher A, Arigoni D, Rohdich F. Biosynthesis of isoprenoids via the non-mevalonate pathway. Cell Mol Life Sci. 2004; 61:1401–1426. 15197467 10.1007/s00018-004-3381-z PMC11138651

[3] Shewmaker CK, Sheehy JA, Daley M, Colburn S, Ke DY. Seed-specific overexpression of phytoene synthase: increase in carotenoids and other metabolic effects. Plant J. 1999; 20: 401–412. 10607293 10.1046/j.1365-313x.1999.00611.x

[4] Ye Z-W, Jiang J-G, Wu G-H. Biosynthesis and regulation of carotenoids in *Dunaliella*: progresses and prospects. Biotechnol Adv. 2008; 26: 352–360. 10.1016/j.biotechadv.2008.03.004 18486405

[5] Bouvier F, Rahier A, Camara B. Biogenesis, molecular regulation and function of plant isoprenoids. Prog. Lipid Res. 2005; 44: 357–429. 16289312 10.1016/j.plipres.2005.09.003

[6] Prieto A, Canavate JP, Garcia-Gonzalezb M. Assessment of carotenoid production by *Dunaliella salina* in different culture systems and operationregimes. J. Biotechnol. 2011; 151: 180–185. 10.1016/j.jbiotec.2010.11.011 21111012

[7] Ben-Amotz A, Polle JE, Rao DS. The alga *Dunaliella*: biodiversity, physiology, genomics and biotechnology. Science Publishers Enfield, NH; 2009.

[8] Rabbani S, Beyer P, Lintig J, Hugueney P, Kleinig H. Induced beta-carotene synthesis driven by triacylglycerol deposition in the unicellular alga *Dunaliella bardawil*. Plant Physiol. 1998; 116: 1239–1248. 9536040 10.1104/pp.116.4.1239 PMC35030

[9] Capa-Robles W, Paniagua-Michel J, Soto JO. The biosynthesis and accumulation of beta-carotene in *Dunaliella salina* proceed via the glyceraldehyde 3-phosphate/pyruvate pathway. Nat Prod Res. 2009; 23: 1021–1028. 10.1080/14786410802689689 19521917

[10] Paniagua-Michel J, Capa-Robles W, Olmos-Soto J, Gutierrez-Millan LE. The carotenogenesis pathway via the isoprenoid-beta-carotene interference approach in a new strain of *Dunaliella salina* isolated from Baja California Mexico. Mar Drugs. 2009; 7: 45–56. 10.3390/md7010045 19370170 PMC2666888

[11] Nikookar K, Moradshahi A, Hosseini L. Physiological responses of *Dunaliella salina* and *Dunaliella tertiolecta* to copper toxicity. Biomol Eng. 2005; 22: 141–146. 16103009 10.1016/j.bioeng.2005.07.001

[12] Ramos AA, Marques AR, Rodrigues M, Henriques N, Baumgartner A, Castilho R, et al Molecular and functional characterization of a cDNA encoding 4-hydroxy-3-methylbut-2-enyl diphosphate reductase from *Dunaliella salina*. J Plant Physiol. 2009; 166: 968–977. 10.1016/j.jplph.2008.11.008 19155093

[13] Sanchez-Estudillo L, Freile-Pelegrin Y, Rivera-Madrid R, Robledo D, Narvaez-Zapata JA. Regulation of two photosynthetic pigment-related genes during stress-induced pigment formation in the green alga, *Dunaliella salina*. Biotechnol Lett. 2006; 28: 787–791. 16786242 10.1007/s10529-006-9001-2

[14] Coesel SN, Baumgartner AC, Teles LM, Ramos AA, Henriques NM, Cancela L, et al Nutrient limitation is the main regulatory factor for carotenoid accumulation and for Psy and Pds steady state transcript levels in *Dunaliella salina* (chlorophyta) exposed to high light and salt stress. Mar Biotechnol. 2008; 10: 602–611. 10.1007/s10126-008-9100-2 18449600

[15] Takahashi H, Kopriva S, Giordano M, Saito K, Hell R. Sulfur assimilation in photosynthetic organisms: molecular functions and regulations of transporters and assimilatory enzymes. Annu Rev Plant Biol. 2011; 62: 157–184. 10.1146/annurev-arplant-042110-103921 21370978

[16] Salguero A, de la Morena B, Vigara J, Vega JM, Vilchez C, León R. Carotenoids as protective response against oxidative damage in *Dunaliella bardawil*. Biomol Eng. 2003; 20: 249–253. 12919805 10.1016/s1389-0344(03)00065-0

[17] Sánchez-Estudillo L, Freile-Pelegrin Y, Rivera-Madrid R, Robledo D, Narváez-Zapata JA. Regulation of two photosynthetic pigment-related genes during stress-induced pigment formation in the green alga, *Dunaliella salina*. Biotechnol Lett. 2006; 28: 787–791. 16786242 10.1007/s10529-006-9001-2

[18] Ramos A, Coesel S, Marques A, Rodrigues M, Baumgartner A, Noronha J, et al Isolation and characterization of a stress-inducible *Dunaliella salina* Lcy-beta gene encoding a functional lycopene beta-cyclase. Appl Microbiol Biotechnol. 2008; 79: 819–828. 10.1007/s00253-008-1492-4 18461318

[19] Lamers PP, Janssen M, De Vos RC, Bino RJ, Wijffels RH. Carotenoid and fatty acid metabolism in nitrogen-starved *Dunaliella salina*, a unicellular green microalga. J Biotechnol. 2012; 162: 21–27. 10.1016/j.jbiotec.2012.04.018 22750089

[20] Cao H, Zhang L, Melis A. Bioenergetic and metabolic processes for the survival of sulfur-deprived *Dunaliella salina* (chlorophyta). J Appl Phycol. 2001; 13: 25–34.

[21] Tran D, Doan N, Louime C, Giordano M, Portilla S. Growth, antioxidant capacity and total carotene of *Dunaliella salina* DCCBC15 in a low cost enriched natural seawater medium. World J Microbiol Biotechnol. 2013; 30: 317–322. 10.1007/s11274-013-1413-2 23821128

[22] Kichtenthaler H, Wellburn A. Determinations of total carotenoids and chlorophyll *a* and *b* of leaf extracts in different solvent. Biochem Soc Trans. 1983; 603: 591–593.

[23] Saha SK, Hayes J, Moane S, Murray P. Tagging of biomolecules with deuterated water (D2O) in commercially important microalgae. Biotechnol Lett. 2013; 35: 1067–1072. 10.1007/s10529-013-1176-8 23479414

[24] Chen H, Jiang J-G. Toxic effects of chemical pesticides (trichlorfon and dimehypo) on *Dunaliella salina*. Chemosphere. 2011; 84:664–670. 10.1016/j.chemosphere.2011.03.032 21621243

[25] Scherer S. Do photosynthetic and respiratory electron transport chains share redox proteins? Trends Biochem Sci. 1990; 15: 458–462. 1963954 10.1016/0968-0004(90)90296-n

[26] Johnson MK, Johnson EJ, MacElroy RD, Speer HL, Bruff BS. Effects of salts on the halophilic alga *Dunaliella viridis*. J Bacteriol. 1968; 95: 1461–1468. 5646631 10.1128/jb.95.4.1461-1468.1968 PMC315107

[27] García-González M, Moreno J, Manzano JC, Florencio FJ, Guerrero MG. Production of *Dunaliella salina* biomass rich in 9-cis-*β*-carotene and lutein in a closed tubular photobioreactor. J Biotechnol. 2005; 115: 81–90. 15607227 10.1016/j.jbiotec.2004.07.010

[28] Hosseini TA, Shariati M. *Dunaliella* biotechnology: methods and applications. J Appl Microbiol. 2009; 107: 14–35. 10.1111/j.1365-2672.2009.04153.x 19245408

[29] Fu W, Paglia G, Magnúsdóttir M, Steinarsdóttir EA, Gudmundsson S, Palsson BO, et al Effects of abiotic stressors on lutein production in the green microalga *Dunaliella salina*. Microb Cell Fact. 2014; 13:1–9. 24397433 10.1186/1475-2859-13-3 PMC3893366

[30] Okamoto O, Pinto E, Latorre L, Bechara E, Colepicolo P. Antioxidant modulation in response to metal-induced oxidative stress in algal chloroplasts. Arch Environ Con Tox. 2001; 40: 18–24. 10.1007/s002440010144 11116337

[31] Shaish A, Avron M, Pick U, Ben-Amotz A. Are active oxygen species involved in induction of *β*-carotene in *Dunaliella bardawil*? Planta. 1993; 190: 363–368.

[32] Rabinowitch HD, Privalle CT, Fridovich I. Effects of paraquat on the green alga *Dunaliella salina*: protection by the mimic of superoxide dismutase, Desferal-Mn (IV). Free Radic Biol Med. 1987; 3: 125–131. 3666516 10.1016/s0891-5849(87)80007-2

[33] Yang XJ, Finnegan PM. Regulation of phosphate starvation responses in higher plants. Ann Bot. 2010; 105: 513–526. 10.1093/aob/mcq015 20181569 PMC2850799

[34] Schachtman DP, Reid RJ, Ayling SM. Phosphorus uptake by plants: from soil to cell. Plant Physiol. 1998; 116: 447–453. 9490752 10.1104/pp.116.2.447 PMC1539172

[35] Kolber Z, Zehr J, Falkowski P. Effects of growth irradiance and nitrogen limitation on photosynthetic energy conversion in photosystem II. Plant Physiol. 1988; 88: 923–929. 16666405 10.1104/pp.88.3.923 PMC1055683

[36] Sano Y, Endo K, Tomo T, Noguchi T. Modified molecular interactions of the pheophytin and plastoquinone electron acceptors in photosystem II of chlorophyll d-containing Acaryochloris marina as revealed by FTIR spectroscopy. Photosynth Res. 2015; 125: 105–114. 10.1007/s11120-014-0073-x 25560630

[37] Lers A, Biener Y, Zamir A. Photoinduction of Massive beta-Carotene Accumulation by the Alga Dunaliella bardawil: Kinetics and Dependence on Gene Activation. Plant Physiol. 1990; 93: 389–395 16667478 10.1104/pp.93.2.389 PMC1062523

