# Supplementary material for: Analysis of the Physiological and Molecular Responses of Dunaliella salina to Macronutrient Deprivation
Source: PLoS One. 2016 Mar 29;11(3):e0152226. doi: 10.1371/journal.pone.0152226 (PMC4811551; doi:10.1371/journal.pone.0152226)
Supplement: S1 Table — (DOCX) [file pone.0152226.s001.docx]

S1 Table. Components of modified Johnson’s medium

| Components | Per liter |
| --- | --- |
| NaCl | 60 g |
| MgCl_2_ | 0.594g |
| MgSO_4_ | 0.244 g |
| KCl | 0.2 g |
| CaCl_2_ | 0.035 g |
| KNO_3_ | 0.5 g |
| NaHCO_3_ | 0.151 g |
| KH_2_PO_4_ | 0.043 g |
| Na_2_EDTA | 1.89 mg |
| FeC1_3_ | 1.47 mg |
| ZnCl_2_ | 41 μg |
| H_3_BO_3_ | 0.61 mg |
| CoCl_2_ | 28 μg |
| CuSO_4_ | 38 μg |
| MnCl_2_ | 26 μg |
| (NH4)_6_Mo_7_O_24_ | 0.36 mg |
