# Supplementary material for: Analysis of the Physiological and Molecular Responses of Dunaliella salina to Macronutrient Deprivation
Source: PLoS One. 2016 Mar 29;11(3):e0152226. doi: 10.1371/journal.pone.0152226 (PMC4811551; doi:10.1371/journal.pone.0152226)
Supplement: S2 Table — (DOCX) [file pone.0152226.s002.docx]

S2 Table. Primers used in qRT-PCR.

| Gene | Accession no | Primer |
| --- | --- | --- |
| 18S rRNA | KF825550 | 5' GGGTGTGCTGGTGAAGTGTTT 3'; 5' CCTATCATCCGCAAGTGAAGTAT 3' |
| DXS | FJ469276 | 5' ACACCAACTACTTTGCGGACTC 3'; 5' CAATGCCGACATCAAACACC 3' |
| HDR | JQ762450 | 5' GCACAAGCTGCACTACGGG 3'; 5' ATCCTTCATGCGGAACACG 3' |
| ZDS | HM754265 | 5' TGAGCAAGGTGGCACTGAA 3'; 5' GGTAGCACCCGAAGAAGACG 3' |
| LCYB | EU327876 | 5' GGATCTGCGCCCGTTGTT 3'; 5' CAGCGGCAGCCATTTCCT 3' |
| CHYB | JN118489 | 5' CCCATCCCAGCAACAGCAG 3'; 5' TGGCAAGCCCAGTTACGC 3' |
| PSY | DBU91900 | 5'GCATGCATCCACAGGCCAACACAGG3';5'TTCACGTCCAAGCAGTCCACCCCATCC3' |
| PDS1 | GQ923693 | 5' ACATGCAGCGGCTGTTTAAG 3'; 5' CATCCTGCTCCTCCACATACTTCT 3' |
| PDS2 | AY954517 | 5' GCACGCATCAAGCAAATCG 3'; 5' TACGCATCTCCCTCAACAATC 3' |
| PDS3 | Y14807 | 5' TCACGCTTTGAGTTCCCAGAC 3'; 5' ATCCTGCTCCTCCACATACTTTT 3' |
